# Outcomes of Neoadjuvant Chemotherapy for Invasive Intraductal Papillary Mucinous Neoplasm Compared with de Novo Pancreatic Adenocarcinoma

**DOI:** 10.1245/s10434-023-14875-5

**Published:** 2024-02-06

**Authors:** Alessandro Fogliati, Andrea Zironda, Guido Fiorentini, Stella Adjei, Abdelrahman Amro, Patrick P. Starlinger, Travis E. Grotz, Susanne G. Warner, Rory L. Smoot, Cornelius A. Thiels, Michael L. Kendrick, Sean P. Cleary, Mark J. Truty

**Affiliations:** 1 Division of Hepatobiliary and Pancreas Surgery, Mayo Clinic, Rochester, MN USA; 2 Department of Medicine and Surgery, University of Milano Bicocca, Milan, Italy; 3 Department of Medicine and Surgery, University of Milano Statale, Milan, Italy; 4 Department of Surgery, University of Toronto, Toronto, ON Canada

**Keywords:** IPMN, PDAC, Neoadjuvant therapy, Pancreatic cancer, Pancreatic cyst, Pancreatic surgery, Pancreatic ductal adenocarcinoma, Intraductal papillary mucinous cystic neoplasm

## Abstract

**Background:**

The management of invasive intraductal papillary mucinous cystic neoplasm (I-IPMN) does not differ from de novo pancreatic ductal adenocarcinoma (PDAC); however, I-IPMNs are debated to have better prognosis. Despite being managed similarly to PDAC, no data are available on the response of I-IPMN to neoadjuvant chemotherapy.

**Methods:**

All patients undergoing pancreatic resection for a pancreatic adenocarcinoma from 2011 to 2022 were included. The PDAC and I-IPMN cohorts were compared to evaluate response to neoadjuvant therapy (NAT) and overall survival (OS).

**Results:**

This study included 1052 PDAC patients and 105 I-IPMN patients. NAT was performed in 25% of I-IPMN patients and 65% of PDAC patients. I-IPMN showed a similar pattern of pathological response to NAT compared with PDAC (*p* = 0.231). Furthermore, positron emission tomography (PET) response (71% vs. 61%; *p* = 0.447), CA19.9 normalization (85% vs. 76%, *p* = 0.290), and radiological response (32% vs. 37%, *p* = 0.628) were comparable between I-IPMN and PDAC. A significantly higher OS and disease-free survival (DFS) of I-IPMN was denoted by Kaplan–Meier analysis, with a *p*-value of < 0.001 in both plots. In a multivariate analysis, I-IPMN histology was independently associated with lower risk of recurrence and death.

**Conclusions:**

I-IPMN patients have a longer OS and DFS after surgical treatment when compared with PDAC patients. The more favorable oncologic outcome of I-IPMNs does not seem to be related to early detection, as I-IPMN histological subclass is independently associated with a lower risk of disease recurrence. Moreover, neoadjuvant effect on I-IPMN was non-inferior to PDAC in terms of pathological, CA19.9, PET, and radiological response and thus can be considered in selected patients.

**Supplementary Information:**

The online version contains supplementary material available at 10.1245/s10434-023-14875-5.

With the increasing resolution of modern imaging devices, incidental diagnosis of intraductal papillary mucinous cystic neoplasms (IPMN) has become common in clinical practice.^1^ Due to the risk of degeneration to invasive adenocarcinoma, surgical resection of high-risk IPMNs is often indicated. Unfortunately, the presentation of IPMNs ranges from main duct IPMNs with ‘high-risk stigmata’ and a high risk of invasive degeneration, to small secondary duct cysts with no ‘worrisome features’ and a low risk of progression.^2,3^ This high variability of risk in IPMNs makes the correct indication for surgical resection one of the challenges in the management of these tumors.^3-5^ The optimal management of patients affected by an invasive IPMN (I-IPMN) is currently debated. I-IPMNs are a specific type of pancreatic adenocarcinoma arising from an IPMN precursor lesion, and there is controversial evidence of a more favorable oncological outcome of these tumors after surgical resection when compared with de novo pancreatic ductal adenocarcinoma (PDAC).^6,7^ Nonetheless, National Comprehensive Cancer Network (NCCN) guidelines^8,9^ do not acknowledge I-IPMNs as a separate entity from PDAC but limit the mention of IPMNs as precursor lesions for pancreatic cancer. When facing an IPMN with a known invasive component, it is currently recommended that both medical and surgical treatment do not differ from the management of PDACs, but data on the effects of chemotherapy on I-IPMN are scarce.

The aim of this study was to determine the prognosis of resected I-IPMNs compared with de novo PDAC, and to assess the response of I-IPMNs to neoadjuvant chemotherapy compared with de novo PDAC.

## Methods

All consecutive patients with a confirmed pathological diagnosis of PDAC or I-IPMN who underwent pancreatic resection between 1 January 2011 and 30 January 2022 in a high-volume center for pancreatic surgery were included in this study. The differentiation between I-IPMN and de novo PDAC is routinely assessed by the institution pathologists by looking for the presence of an IPMN environment around PDAC. Chart review was performed retrospectively and included demographical characteristics, surgical variables, tumor characteristics, imaging characteristics, and follow-up data. Demographical characteristics included age, sex, tumor histology, Eastern Cooperative Oncology Group (ECOG) and American Society of Anesthesiologists (ASA) score, while surgical variables included type of pancreatic resection, concomitant vascular resection, and Clavien–Dindo grade^10^ of postoperative complications assessed within 90 days from surgery. Tumor characteristics included tumor size, staging according to the 8th edition of the American Joint Committee on Cancer (AJCC) staging system for pancreatic adenocarcinoma, preoperative CA19.9, number of harvested and positive lymph nodes, resection margins, tumor grading, perineural invasion and lymphovascular invasion, and follow-up data included overall survival (OS) and disease-free survival (DFS). Disease recurrence was defined as ‘early recurrence’ when occurring in the first 6 months after surgery.

The primary outcome of this study was pathological response to neoadjuvant therapy (NAT), and was defined according to the guidelines of the College of American Pathologists.^11^ Pathological response score was assigned as follows: complete response (score 0), near complete response (score 1), partial response (score 2), and minimal/no response (score 3). Subjects with scores of 0 or 1 were grouped together as ‘major response’, while scores of 2 or 3 were grouped together as ‘partial/no response’. Secondary outcomes were radiological response as defined by the Response Evaluation Criteria in Solid Tumors (RECIST) criteria,^12^ CA19.9 response defined as normalization of marker level (≤37 UI/mL) after NAT regardless of initial CA19.9 level, and positron emission tomography (PET) response. Non-secretor patients were excluded from the CA19.9 response analysis. PET response was defined as ‘major response’ when tumor fluorodeoxyglucose (FDG) uptake was below hepatic FDG uptake, and similar to the background pancreatic tissue or ‘minor response’ if tumor FDG uptake was persistent or higher than the background.

Data were collected in a Microsoft Excel (Microsoft Corporation, Redmond, WA, USA) database and then transferred to SPSS statistics. Statistical analysis was performed using SPSS statistics version 28.0 (IBM Corporation, Armonk, NY, USA). Continuous variables were tested for normality using the Shapiro–Wilk test. Normally distributed continuous variables were expressed as the mean ± standard deviation and compared using Student’s *t*-test, and non-normally distributed variables were expressed as the median with interquartile range and compared using the Mann–Whitney U test. Categorical variables were expressed as number of events (%) and were compared using the Chi-square test. A univariate and multivariate logistic regression analysis was performed to assess correlation to tumor recurrence, and a univariate and multivariate Cox regression analysis was performed to assess correlation to OS. Variables with a *p*-value < 0.05 at univariate analysis were selected for multivariate analysis. The OS and DFS were compared using Kaplan–Meier curves and log-rank test. A *p*-value of < 0.05 was considered as the cut-off for statistical significance. When data regarding a variable were not available, the patient was excluded from that specific variable analysis.

## Results

### Demographics

This study included 1152 patients who underwent pancreatic resection, 1047 (91%) of whom were patients with de novo PDAC and 105 (9%) were patients with I-IPMN. Ethnicity distribution was 1072 (93%) White, 22 (2%) Hispanic, 18 (2%) Asian, 12 (1%) Arabic, 8 (1%) Indian, 8 (1%) African American, 8 (1%) Native American, and 3 (>1%) ‘other’. Among the I-IPMN cases, 39 (37%) patients were considered high-risk IPMN before resection and did not have a preoperative confirmation of malignancy. The type of resection was pancreaticoduodenectomy for 732 (64%) patients, distal pancreatectomy for 277 patients (24%), and total pancreatectomy for the remaining 143 (12%) patients, with no significant difference in distribution between the two groups. PDAC patients were younger (66 [58–73] years vs. 70 [62–75] years, *p* = 0.003) compared with I-IPMN patients. No statistical difference was observed between PDAC and I-IPMN in terms of biological sex (female: 46% vs. 42%, *p* = 0.473), active use of tobacco (12% vs. 9%, *p* = 0.273), diabetes mellitus (34% vs. 35%, *p* = 0.723), ECOG score ≥1 (33% vs. 27%, *p* = 0.183) and ASA score ≥3 (69% vs. 70%, *p* = 0.779) between the two groups. PDAC patients were more commonly treated with chemotherapy (95% vs. 83%, *p* < 0.001) at any point, and were much more likely to undergo NAT (65% vs. 25%, *p* < 0.001), more frequently required concomitant vascular resection (36% vs. 14%, *p* < 0.001), and had a higher rate of Clavien–Dindo grade III or greater postoperative complications (34% vs. 24%, *p* = 0.028), but maintaining a similar 90-day mortality (4% vs. 3%, *p* = 0.684). The preoperative CA19.9 (157.2 ± 523.1 vs. 120.9 ± 336, *p* = 0.502) did not differ significantly between PDAC and I-IPMN. Data on population characteristics are presented in Table 1.

**Table 1:** Population characteristics

	Total *N*= 1152	PDAC *N*= 1047	I-IPMN *N* = 105	*P* value
Age, median (IQR)	66 (59-73)	66 (58-73)	70 (62-75)	**0.003**
Sex, female	521 (45)	477 (46)	44 (42)	0.473
I-IPMN preoperative diagnosis				
PDAC	–	–	28 (27%)	
I-IPMN	–	–	38 (36%)	
High risk IPMN	–	–	39 (37%)	
Preop CA19.9	154 ± 509.2	157.2 ± 523.1	120.9 ± 336	0.502
Active tobacco use	136 (12)	127 (12)	9 (9)	0.273
Diabetes mellitus	388 (34)	351 (34)	37 (35)	0.723
ECOG ≥1	374 (32)	346 (33)	28 (27)	0.183
ASA ≥3	798 (69)	724 (69)	74 (70)	0.779
Any chemotherapy	1081 (94)	994 (95)	87 (83)	**< 0.001**
Neoadjuvant CT	704 (61)	678 (65)	26 (25)	**< 0.001**
Type of neoadjuvant CT FOLFIRINOX Gemcitabine based Other	560 (49) 126 (11) 18 (2)	543 (52) 118 (11) 17 (2)	17 (16) 8 (8) 1 (1)	0.187
Type of pancreatic resection Total pancreatectomy Pancreaticoduodenectomy Distal pancreatectomy	143 (12) 732 (64) 277 (24)	125 (12) 677 (65) 245 (23)	18 (17) 55 (52) 32 (30)	**0.020**
Vascular resection	396 (34)	381 (36)	15 (14)	**< 0.001**
Clavien Dindo ≥3	380 (33)	355 (34)	25 (24)	**0.028**
90-day mortality	41 (4)	38 (4)	3 (3)	0.684

Bold values indicate statistically significant results (*p* < 0.05)

IQR = Interquartile range, PDAC = pancreatic ductal adenocarcinoma, I-IPMN = Invasive intraductal mucinous cystic neoplasm, ECOG = eastern cooperative oncology group, ASA = American society of anesthesiology, CT = chemotherapy

In the subset of patients who underwent NAT, the PDAC group was composed of 678 patients and the I-IPMN group was composed of 26 patients. In this subgroup of patients, there was only a significant difference in age (64.7 ± 9.6 vs. 69.5 ± 6.3, *p* = 0.006) between PDAC and I-IPMN patients. No significant differences were observed in the type of NAT regimen (*p* = 0.187), the number of NAT cycles (7.8 ± 7 vs. 6.1 ± 3.5, *p* = 0.109), and concomitant vascular resection (46% vs. 31%, *p* = 0.133). The characteristics of the NAT subgroups are presented in Table 1 of the electronic supplementary material (ESM).

### Pathological Characteristics

The PDAC and I-IPMN groups were similarly distributed in regard to pathologic stage (stage I: 46% vs. 50%; stage II: 38% vs. 39%; stage III: 15% vs. 10%; stage IV: 1% vs. 2%; *p* = 0.458). The lymph nodal involvement of the PDAC and I-IPMN groups was similar (N0: 57% vs. 63%; N1: 29% vs. 27%; N2: 14% vs. 10%; *p* = 0.506), with a similar amount of mean harvested lymph nodes (21.5 ± 9.8 vs. 20.2 ± 10.8, *p* = 0.190). The distribution of tumor grading was different between the two groups, with I-IPMN having had more G1 and no G4 tumors (undetermined: 7% vs. 2%; G1: 4% vs. 20%; G2: 55% vs. 44%; G3: 32% vs. 33%; G4: 1% vs. 0%; *p* < 0.001). Perineural invasion was more frequently detected in the PDAC group (45% vs. 29%, *p* = 0.001), while rates of lymphovascular invasion were similar (16% vs. 10%, *p* = 0.151). The rate of positive resection margins were significantly not different (10% PDAC vs. 5% I-IPMN, *p* = 0.089). Among I-IPMNs, 68 (65%) developed from main duct or mixed-type IPMNs, 20 (19%) arose from a branch duct IPMN, and for the remaining 17 (16%), this data was uncertain. Pathological variables are reported in Table 2.

**Table 2:** Pathological characteristics

	Total *N*= 1152	PDAC *N*= 1047	I-IPMN *N* = 105	*P* value
Staging Ia/Ib IIa/IIb III IV	533 (46) 437 (38) 167 (14) 15 (1)	481 (46) 396 (38) 157 (15) 13 (1)	52 (50) 41 (39) 10 (10) 2 (2)	0.458
T stage Complete NAT response T 1 a,b,c T 2 T 3 T 4	65 (6) 269 (23) 482 (42) 308 (27) 28 (2)	64 (6) 232 (22) 450 (43) 273 (26) 28 (3)	1 (1) 37 (35) 32 (30) 35 (33) 0	**< 0.001**
N stage N 0 N 1 N 2	667 (58) 332 (29) 153 (13)	601 (57) 304 (29) 142 (14)	66 (63) 28 (27) 11 (10)	0.506
Metastatic at surgery	15 (1)	13 (1)	2 (2)	0.568
Harvested lymph nodes, mean ± SD	21.4 ± 9.9	21.5 ± 9.8	20.2 ± 10.8	0.190
Pathologic grade Undetermined G1 G2 G3 G4	77 (7) 68 (6) 617 (54) 375 (33) 11 (1)	75 (7) 47 (4) 571 (55) 340 (32) 11 (1)	2 (2) 21 (20) 46 (44) 35 (33) 0	**< 0.001**
IPMN duct involvement Branch duct Main duct/Mixed	–	–	– 20 (19) 85 (81)	
Perineural invasion	503 (44)	473 (45)	30 (29)	**0.001**
Lymphovascular invasion	176 (15)	165 (16)	11 (10)	0.151
R1	108 (9)	103 (10)	5 (5)	0.089

Bold values indicate statistically significant results (*p* < 0.05)

SD = standard deviation, NAT = neoadjuvant therapy, PDAC = pancreatic ductal adenocarcinoma, I-IPMN = invasive intraductal mucinous cystic neoplasm

In the NAT subgroup, the only difference between PDAC and I-IPMN among pathological characteristics was the tumor grading, with similar results when compared with the entire population (undetermined: 10% vs. 4%; G1: 4% vs. 23%; G2: 61% vs. 54%; G3: 24% vs. 19%; G4 0.1% vs. 0%, *p* < 0.001). No statistically significant differences in pathologic stage, rates of node positivity, perineural invasion, and lymphovascular invasion were observed in the NAT subpopulation. The pathological characteristics of the NAT patients is available in detail in ESM Table 2.

### Neoadjuvant Therapy Response

Pathologic response in PDAC and I-IPMN patients treated with NAT was assessed as the primary outcome. There was no statistically significant difference between the two groups (marked response: 29% vs. 19%; partial/no response: 67% vs. 81%; *p* = 0.231), and complete NAT response was similar (9% vs. 4%, *p* = 0.322). Secondary outcomes of response to NAT were also similar between PDAC and I-IPMN: CA19.9 normalization (62% vs. 73%, *p* = 0.293), PET response (64% vs. 64%, *p* = 0.990), and radiological RECIST criteria complete/partial response (37% vs. 32%, *p* = 0.628). No statistical difference was present in the difference of CA19.9, change in standardized uptake value, and change in radiological tumor size between the I-IPMN and PDAC groups. Table 3 contains data on response to adjuvant therapy.

**Table 3:** Response to neoadjuvant therapy assessment

	Total *N* = 704	PDAC *N* = 678	I-IPMN *N* = 26	*P* value
NAT response Marked response Partial/No response Undetermined	203 (29) 367 (52) 22 (3)	198 (29) 354 (67) 22 (3)	5 (19) 13 (81) 0	0.231
Complete NAT response	65 (9)	64 (9)	1 (4)	0.322
CA19.9 response	397/640 (62)	381/618 (62)	16/22 (73)	0.293
Δ in CA19-9	−65 (4–301)	−68 (5–305)	−21.5 (0–130)	0.067
PET response	252/393 (64)	243/379 (64)	9/14 (64)	0.990
Δ in SUV	−2.5 (0.1–4.8)	−2.5 (0.1–4.8)	−3.5 (1.5–4.9)	0.206
RECIST criteria Complete response Partial response Stable disease Progressive disease	19 (3) 218 (31) 363 (52) 48 (7)	19 (3) 210 (31) 347 (51) 47 (7)	0 8 (31) 16 (62) 1 (4)	0.685
RECIST complete/partial response	237/648 (37)	229/623 (37)	8/25 (32)	0.628
Δ in radiological size	−5 (0–12)	−5 (0–13)	−5 (0–10)	0.732

NAT = neoadjuvant therapy, PDAC = pancreatic ductal adenocarcinoma, I-IPMN = invasive intraductal mucinous cystic neoplasm, PET = positron emission tomography

### Oncological Outcomes

**Fig. 1:**
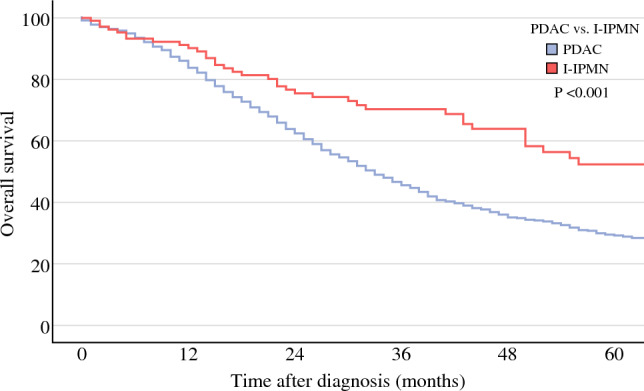
Kaplan–Meier plot for overall survival from date of diagnosis

**Fig. 2:**
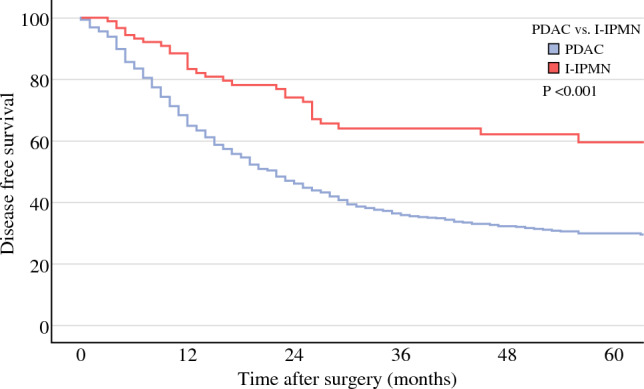
Kaplan–Meier plot for disease-free survival from surgical resection

The median follow-up of the studied cohort was 25 months (interquartile range 14–43). The 5-year OS from date of diagnosis of PDAC patients was 29% compared with 52% of I-IPMN patients (*p* < 0.001) (Fig. 1). Similarly, DFS from surgical resection was lower in the PDAC patients when compared with I-IPMN patients (5-year DFS: 30% vs. 60%, *p* < 0.001) (Fig. 2). When analyzed by tumor stage, I-IPMN vs. PDAC OS was significantly different for stage I and stage II disease (stage I: 66% vs. 41% [*p* < 0.001]; stage II: 50% vs. 23% [*p* < 0.011], and stage III: 0% vs. 8% [*p* = 0.536]). A visual representation of OS by oncological stage in PDAC and I-IPMN patients is available in ESM Fig. 1. The number of early recurrence events were lower in the I-IPMN group (14% vs. 6%, *p* = 0.014). The observed pattern of recurrence showed a higher percentage of lung (*n* = 132/530 [13%] vs. *n* = 4/31 [4%], *p* = 0.008), liver (*n* = 176/530 [17%] vs. *n* = 9/31 [9%], *p* = 0.030), and peritoneal (*n* = 92/530 [9%] vs. *n* = 3/31 [3%], *p* = 0.037) recurrence in PDAC.

**Fig. 3:**
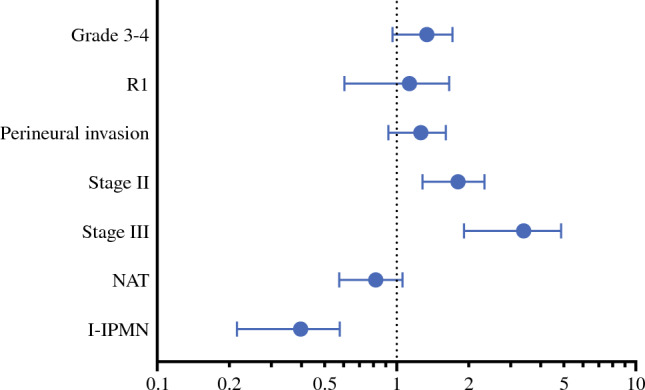
Multivariate binary regression analysis for disease recurrence on the forest plot

After an initial univariate analysis for variables associated with tumor recurrence (ESM Table 3), tumor grading, resection margins, perineural invasion, staging, NAT, and I-IPMN were selected for a multivariate binary logistic regression (Table 4). The results obtained are presented on the forest plot in Fig. 3. Grade 3 and 4 tumors (odds ratio [OR] 1.3, confidence interval [CI] 0.98–1.73, *p* = 0.068), positive resection margins (OR 1.05, CI 0.65–1.69, *p* = 0.838), patients who underwent NAT (OR 0.79, CI 0.59–1.07, *p* = 0.122) and perineural invasion (OR 1.23, CI 0.94–1.62, *p* = 0.129) did not significantly correlate with disease recurrence at multivariate analysis, even though tumor grading is close to a *p*-value of <0.05. Disease staging according to AJCC 8th edition was observed as a significant and independent predictor of disease recurrence; stage II disease had an OR of 1.75 (CI 1.31–2.35, *p* < 0.001), while stage III had an OR of 3.18 (CI 2.03–4.97, *p* < 0.001). At last, I-IPMN subtype of pancreatic cancer was strongly associated with a reduced chance of disease recurrence, with an OR of 0.35 (CI 0.22–0.57, *p* < 0.001).

**Table 4:** Multivariate logistic regression analysis for disease recurrence

Multivariate binary logistic regression: disease recurrence	OR	CI	*P* value
Grade			
1–2 (ref)	–	–	–
3–4	1.3	0.98–1.73	0.068
R1	1.05	0.65–1.69	0.838
Perineural invasion	1.23	0.94–1.62	0.129
Staging			
Ia/Ib (ref)	–	–	–
IIa/IIb	1.75	1.31–2.35	**<0.001**
III	3.18	2.03–4.97	**<0.001**
NAT	0.79	0.59–1.07	0.122
I-IPMN	0.37	0.23–0.59	**<0.001**

Multivariate Cox regression: survival	HR	CI	P value
Grade			
1–2 (ref)	–	–	–
3–4	1.37	1.17–1.62	**<0.001**
R1	1.2	0.95–1.53	0.135
Perineural invasion	0.98	0.83–1.15	0.805
Staging			
Ia/Ib (ref)	–	–	–
IIa/IIb	1.61	1.33–1.94	**<0.001**
III	2.64	2.09–3.32	**<0.001**
NAT	0.83	0.7–0.99	**0.041**
I-IPMN	0.55	0.39– 0.76	**<0.001**
Vascular resection	1.34	1.13–1.59	**<0.001**

Bold values indicate statistically significant results (*p* < 0.05)

NAT = neoadjuvant therapy, I-IPMN = invasive intraductal mucinous cystic neoplasm, OR = odds ratio, HR = hazard ratio, CI = confidence interval

After univariate Cox regression analysis for variables associated with survival (ESM Table 3), tumor grading, resection margins, perineural invasion, staging, NAT, vascular resection and I-IPMN were selected for multivariate Cox regression (Table 4). The results are presented on a forest plot in ESM Fig. 2. Resection margins (hazard ratio [HR] 1.2, CI 0.95–1.53, *p* = 0.135) and perineural invasion (HR 0.98, CI 0.83–1.15, *p* = 0.805) did not reach statistical significance. Grade 3 and 4 tumors had an HR of 1.37 (CI 1.17–1.62, *p* < 0.001), vascular resection had an HR of 1.34 (CI 1.13–1.59, *p* < 0.001), and stage II (HR 1.61, CI 1.33–1.94, *p* < 0.001) and III (HR 2.64, CI 2.1–3.32, *p* < 0.001) had a positive HR. Patients who underwent NAT had an HR of 0.83 (CI 0.7–0.99, *p* < 0.001), while patients with an I-IPMN had an HR of 0.55 (CI 0.39–0.79, *p* < 0.001).

## Discussion

The present study involves a large cohort of resected pancreatic cancer patients who were treated in a high-volume tertiary center for pancreatic surgery. I-IPMNs accounted for 9% of surgical resection for PDAC, which is similar to prior reported rates.^13^ The aim of this study was to compare the OS and the response to NAT of I-IPMN and PDAC. In the observed cohorts we observed a significantly longer OS of I-IPMN, however no differences in pathological, CA19.9, PET, and radiological response to NAT between I-IPMN and PDAC was observed.

From the demographical data observed in this study, there are some differences in the average I-IPMN and de novo PDAC populations. I-IPMN patients tend to be older, they undergo a distal and total pancreatectomy slightly more often, they are less likely to need a vascular resection, they receive NAT less often, and they have fewer major postoperative complications. The increased age of I-IPMN patients might be the consequence of a slow process of malignant degeneration.^14^ The higher proportion of total pancreatectomies is instead probably to be attributed to the nature of these pancreatic cystic neoplasms, as they can present with an involvement of the whole gland. As I-IPMN patients were less often undergoing a pancreaticoduodenectomy and vascular resection,^15^ they also had fewer major postoperative complications, but the 90-day mortality after surgery did not differ. The lower use of NAT in I-IPMN patients can be partially explained by the 37% preoperative uncertainty of malignancy and the lower proportion of tumor with vascular involvement, but even by accounting for these patients it appears that in current clinical practice I-IPMNs with a known malignancy are more likely to receive upfront surgery, even though current medical oncology guidelines do not mention a different approach to I-IPMN and PDAC.^8,9^ The role of NAT in I-IPMN has not been thoroughly investigated and currently available literature on the subject is scarce; however, in a large cohort of 240 resected I-IPMN patients, it was reported that NAT was used in only 2.5% of cases.^16^ Regarding histological differences between the two cohorts, I-IPMNs had more well-differentiated (G1) tumors, more T1 tumors, and were less likely to invade perineurally. Nonetheless, the nodal involvement was similar and the overall staging distribution of I-IPMN and PDAC did not differ significantly.

The role of adjuvant chemotherapy in I-IPMN has recently been discussed, with several retrospective studies^17-19^ suggesting that it might not improve OS in the absence of nodal involvement. Unfortunately, as only a minority of patients with an I-IPMN undergo NAT, the I-IPMN cohort was small and no strong recommendations are possible on the basis of this study. Despite the small statistical power of this study, to our knowledge, this is the first study to assess the response of I-IPMN to NAT and to compare it with de novo PDAC. This makes the study a relevant first step in understanding the topic. In light of our observations, the scarce use of NAT in I-IPMN might not be justified. In the case of preoperative confirmation of malignancy with fine needle aspiration, the use of NAT in I-IPMN could provide the same advantages that it provides in PDAC.^20,21^ This is particularly true when radiological suspicion of nodal involvement is present at preoperative disease staging, as adjuvant therapy has been proven beneficial in these settings.^17-19^

A secondary result obtained from this study is the suggestion that I-IPMNs are not the same disease as de novo PDAC. Similar to other observations in the literature,^22^ I-IPMNs have been associated with a more favorable outcome than PDAC, with a higher OS and DFS. Another indicator of a less aggressive biology was the lower rate of early recurrence in the first 6 months after surgery. Moreover, at multivariate analysis, the I-IPMN subtype of pancreatic cancer was an independent predictor of both lower risk of recurrence and death.

The retrospective nature of this study and the small cohort of I-IPMNs undergoing NAT make it hard to draw strong conclusions. Selection and time bias may have influenced treatment decisions, including the use of NAT in I-IPMN. The small size of the I-IPMN cohort may have led our analysis to be underpowered to detect clinically significant differences in some comparisons with PDAC.

## Conclusion

Pancreatic adenocarcinoma arising from an IPMN is independently associated with longer OS and DFS after surgical treatment when compared with de novo PDAC. Patients with I-IPMNs are less likely to receive NAT before surgical resection, but when undergoing NAT, they have a similar response compared with patients affected by de novo PDAC. These data show that the potential efficacy of NAT on I-IPMN is similar to that of PDAC and thus may be considered in selected high-risk patients with I-IPMN similar to PDAC indications.

### Supplementary Information

Below is the link to the electronic supplementary material.

Supplementary file1 (DOCX 381 KB)
