# Supplementary material for: Outcomes of Neoadjuvant Chemotherapy for Invasive Intraductal Papillary Mucinous Neoplasm Compared with de Novo Pancreatic Adenocarcinoma
Source: Ann Surg Oncol. 2024 Feb 6;31(4):2632–9. doi: 10.1245/s10434-023-14875-5 (PMC10908613; doi:10.1245/s10434-023-14875-5)

Supplementary Table 1 - Population characteristics in neoadjuvant subset

|  | Total  N=704 | PDAC  N= 678 | I-IPMN  N= 26 | P value |
| --- | --- | --- | --- | --- |
| Age, years, median (IQR) | 65 (58-72) | 65 (58-72) | 70.5 (65-74) | **0.006** |
| Sex, female | 328 (47) | 314 (46) | 14 (54) | 0.450 |
| Active tobacco use | 67 (10) | 64 (9) | 3 (12) | 0.730 |
| Diabetes mellitus | 254 (37) | 247 (36) | 7 (27) | 0.322 |
| ECOG ≥1 | 259 (37) | 251 (37) | 8 (31) | 0.517 |
| ASA ≥3 | 518 (74) | 500 (74) | 18 (69) | 0.608 |
| Neoadjuvant CT   - *FOLFIRINOX* - *Gemcitabine based* - *Other* | 560 (80)  126 (18)  18 (3) | 543 (80)  118 (17)  17 (3) | 17 (65)  8 (31)  1 (4) | 0.187 |
| Neoadjuvant cycles, mean ± SD | 7.8 ± 6.9 | 7.8 ± 7 | 6.1 ± 3.5 | 0.109 |
| Type of pancreatic resection   - *Total pancreatectomy* - *Pancreaticoduodenectomy* - *Distal pancreatectomy* | 112 (16)  435 (62)  157 (22) | 108 (16)  420 (62)  150 (22) | 4 (15)  15 (58)  7 (27) | 0.975 |
| Vascular resection | 318 (45) | 310 (46) | 8 (31) | 0.133 |
| Clavien Dindo ≥3 | 285 (40) | 278 (41) | 7 (27) | 0.129 |
| 90-day mortality | 32 (5) | 31 (5) | 1 (4) | 0.862 |

IQR=Interquartile range, PDAC= pancreatic ductal adenocarcinoma, I-IPMN= Invasive intraductal mucinous cystic neoplasm, ECOG= eastern cooperative oncology group, ASA= American society of anesthesiology, CT= chemotherapy.

Supplementary Table 2 - Pathological characteristics in neoadjuvant subset

|  | Total  N=704 | PDAC  N= 678 | I-IPMN  N= 26 | P value |
| --- | --- | --- | --- | --- |
| Staging  *Ia/Ib*  *IIa/IIb*  *III*  *IV* | 415 (59)  218 (31)  62 (9)  9 (1) | 403 (59)  207 (31)  59 (9)  9 (1) | 12 (46)  11 (42)  3 (12)  0 | 0.355 |
| T stage  *Complete NAT response*  *T 1 a,b,c*  *T 2*  *T 3*  *T 4* | 65 (9)  209 (30)  279 (40)  127 (18)  24 (3) | 64 (9)  201 (30)  270 (40)  119 (18)  24 (4) | 1 (4)  8 (31)  9 (35)  8 (31)  0 | 0.358 |
| N stage  *N 0*  *N 1*  *N 2* | 502 (71)  153 (22)  49 (7) | 487 (72)  145 (21)  46 (7) | 15 (58)  8 (31)  3 (12) | 0.282 |
| Harvested lymphnodes, mean ± SD | 21.3 ± 10.2 | 21.3 ± 10.1 | 22.2 ± 11.2 | 0.315 |
| Grading  *Undetermined*  *G1*  *G2*  *G3*  *G4* | 72 (10)  31 (4)  430 (61)  169 (24)  1 (0.1) | 71 (10)  25 (4)  416 (61)  164 (24)  1 (0.1) | 1 (4)  6 (23)  14 (54)  5 (19)  0 | **<0.001** |
| Perineural invasion | 88 (13) | 83 (12) | 5 (19) | 0.290 |
| Lymphovascular invasion | 301 (43) | 292 (43) | 9 (35) | 0.393 |
| R1 | 46 (7) | 45 (7) | 1 (4) | 0.572 |

SD=standard deviation, PDAC= pancreatic ductal adenocarcinoma, I-IPMN= invasive intraductal mucinous cystic neoplasm.

Supplementary table 3 – Univariate logistic regression for disease recurrence

| Univariate binary logistic regression: disease recurrence | OR | CI | P value |
| --- | --- | --- | --- |
| NAT | 0.64 | 0.5 – 0.83 | **<0.001** |
| Staging  *Ia/Ib (ref)*  *IIa/IIb*  *III* | –  2.24  4.2 | –  1.71 – 2.92  2.76 – 6.38 | –  **<0.001**  **<0.001** |
| Grade  *1-2 (ref)*  *3-4* | –  1.44 | –  1.10 – 1.88 | –  **0.007** |
| Perineural invasion | 1.65 | 1.3 – 2.11 | **<0.001** |
| ECOG ≥1 | 0.93 | 0.72 – 1.2 | 0.549 |
| R1 | 1.59 | 1.02 – 2.49 | **0.043** |
| N staging  *N0 (ref)*  *N1*  *N2* | –  1.79  4.05 | –  1.36 – 2.36  2.6 – 6.31 | –  **<0.001**  **<0.001** |
| I-IPMN | 0.4 | 0.25 – 0.62 | **0.011** |
| Vascular resection | 0.86 | 0.67 – 1.12 | 0.241 |
| Univariate Cox regression: Survival | **HR** | **CI** | **P value** |
| NAT | 0.74 | 0.64 – 0.86 | **<0.001** |
| Staging  *Ia/Ib (ref)*  *IIa/IIb*  *III* | –  1.79  3.30 | –  1.51 – 2.13  2.59 – 3.97 | –  **<0.001**  **<0.001** |
| Grade  *1-2 (ref)*  *3-4* | –  1.43 | –  1.22 – 1.67 | –  **<0.001** |
| Perineural invasion | 1.26 | 1.08 – 1.46 | **0.003** |
| ECOG ≥1 | 1.11 | 0.95 – 1.31 | 0.196 |
| R1 | 1.6 | 1.27 – 2.01 | **<0.001** |
| N staging  *N0 (ref)*  *N1*  *N2* | –  1.63  2.92 | –  1.38 – 1.93  2.37 – 3.59 | –  **<0.001**  **<0.001** |
| I-IPMN | 0.51 | 0.37 – 0.7 | **<0.001** |
| Vascular resection | 1.24 | 1.06 – 1.45 | **0.008** |

IQR=Interquartile range, PDAC= pancreatic ductal adenocarcinoma, I-IPMN= Invasive intraductal mucinous cystic neoplasm, ECOG= eastern cooperative oncology group, ASA= American society of anesthesiology, CT= chemotherapy, NAT= Neoadjuvant therapy.

Supplementary figure 1 – Kaplan-Meier plot for overall survival of PDAC and I-IPMN by stage


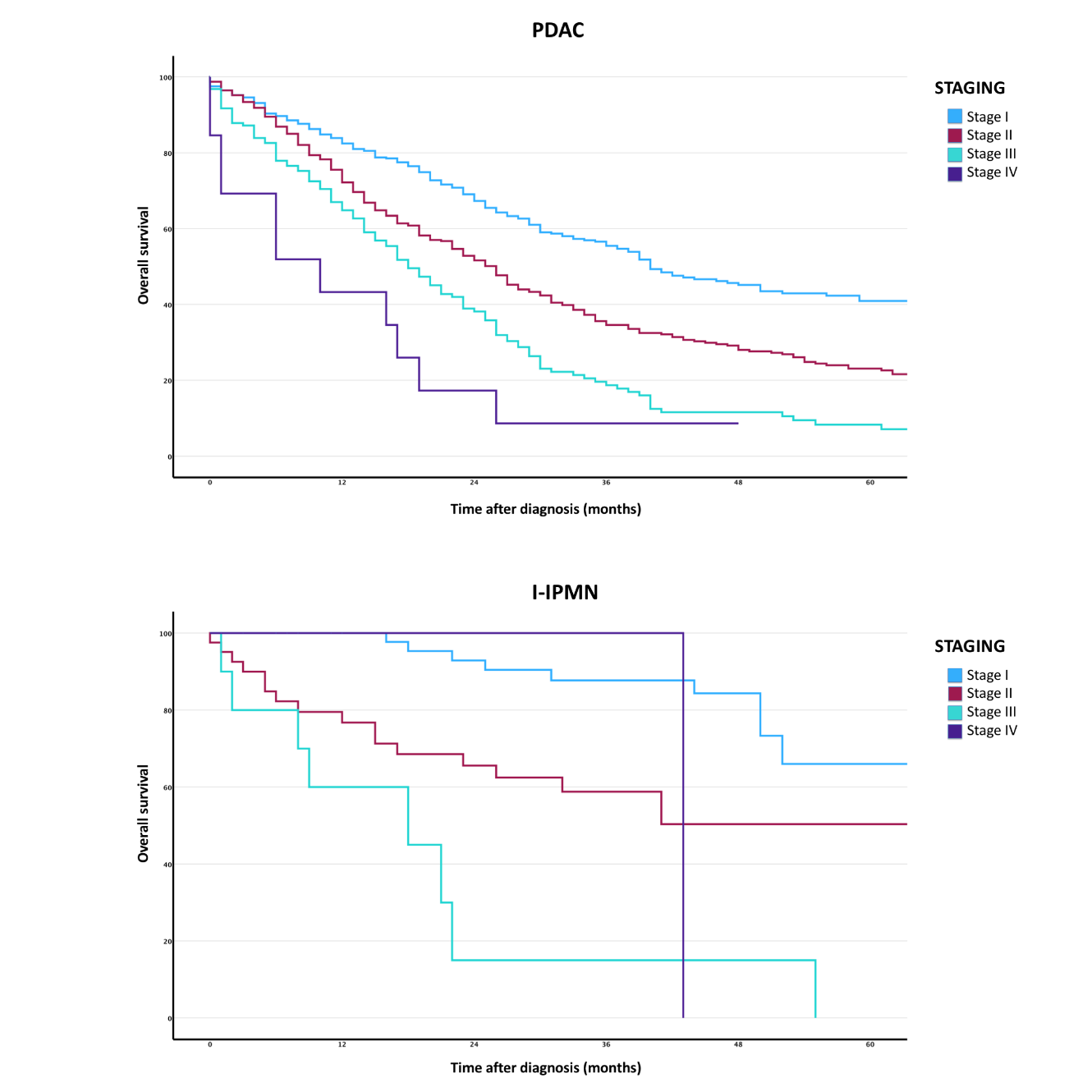


Supplementary figure 2 – Multivariate Cox regression analysis for survival on Forest plot


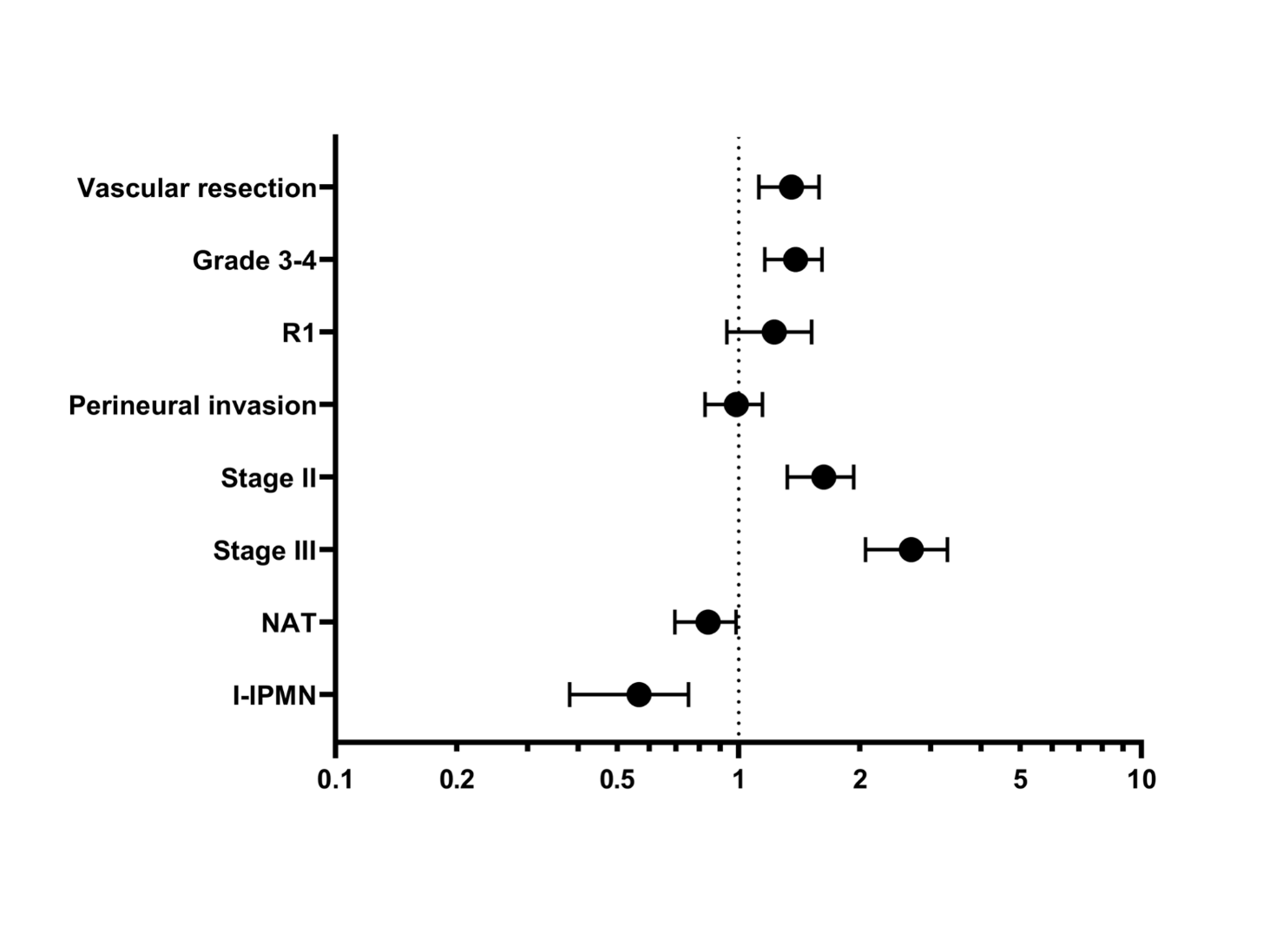

Supplement: Supplementary file 1 — Supplementary file1 (DOCX 381 KB) [file 10434_2023_14875_MOESM1_ESM.docx]
